# Surveillance of tick-borne pathogens in domestic dogs from Chad, Africa

**DOI:** 10.1186/s12917-024-04267-6

**Published:** 2024-09-18

**Authors:** Ellen Haynes, Kayla B. Garrett, Ryan K. A. Grunert, John A. Bryan, Metinou Sidouin, Philip Tchindebet Oaukou, Bongo Nare Richard Ngandolo, Michael J. Yabsley, Christopher A. Cleveland

**Affiliations:** 1 Southeastern Cooperative Wildlife Disease Study, Department of Population Health, College of Veterinary Medicine, Wildlife Health Building, 589 D.W, Brooks Dr, Athens, GA 30602 USA; 2 Warnell School of Forestry and Natural Resources, University of Georgia, Athens, GA 30602 USA; 3 Zachery Consulting, LLC, 2595 Rogers Mill Road, Danielsville, GA 30633 USA; 4 The Carter Center, National Guinea Worm Eradication Program, BP 440, N’Djamena, Chad; 5 Programme National d’Eradication du Ver de Guinée, Ministry of Health, N’Djamena, Chad; 6 Institut de Recherche en Elevage pour le Développement, Afrique One Aspire, N’Djamena, Chad; 7 Center for Ecology of Infectious Diseases, University of Georgia, 203 D.W. Brooks Drive, Athens, GA 30602 USA

**Keywords:** Africa, Canine health, Tick-borne diseases, Zoonoses

## Abstract

**Background:**

Tick-borne pathogens are understudied among domestic animals in sub-Saharan Africa but represent significant threats to the health of domestic animals and humans. Specifically, additional data are needed on tick-borne pathogens in Chad, Africa. Surveillance was conducted among domestic dogs in Chad for selected tick-borne pathogens to measure (1) the prevalence of antibodies against *Anaplasma* spp., *Borrelia burgdorferi*, and *Ehrlichia* spp.; (2) the prevalence of infections caused by *Hepatozoon* spp., *Ehrlichia canis*, *Anaplasma platys*, and *Babesia* spp.; and (3) associations of pathogens with demographic, spatial, and temporal factors. Blood samples were collected from domestic dogs at three time points (May 2019, November 2019, June 2020) across 23 villages in southern Chad.

**Results:**

Of the 428 dogs tested with the IDEXX SNAP 4Dx test in May 2019, 86% (*n* = 370, 95% CI = 83–90%) were positive for antibodies to *Ehrlichia* spp., 21% (*n* = 88, 95% CI = 17–25%) were positive for antibodies to *Anaplasma* spp., and 0.7% (*n* = 3, 95% CI = 0.1–2%) were positive for antibodies to *Borrelia burgdorferi*. Four different pathogens were detected via PCR. *Hepatozoon* spp. were most commonly detected (67.2–93.4%, depending on the time point of sampling), followed by *E. canis* (7.0-27.8%), *A. platys* (10.1–22.0%), and *Babesia vogeli* (0.4–1.9%). Dogs were coinfected with up to three pathogens at a single time point, and coinfections were most common in May 2019 compared to November 2019 and May 2020.

**Conclusions:**

Overall, this study provides new data about the epidemiology of tick-borne pathogens in domestic dogs in Chad, with potential implications for dog and human health.

## Background

Vector-borne diseases, especially those caused by tick-borne pathogens, are a significant health concern for humans and domestic animals in sub-Saharan Africa and should be studied in the context of One Health [1]. Although tick-borne pathogens are widespread in Africa, there are still considerable knowledge gaps regarding pathogen prevalence, vectors, geographic distribution, and host ranges. Furthermore, changes in climate and habitat alter the distributions of ticks and their pathogens, resulting in the introduction of ticks and tick-borne pathogens into novel areas with naïve host populations [2]. Populations of domestic dogs can harbor a high prevalence and diversity of pathogens that can cause morbidity and mortality, and because some of these tick-borne pathogens are zoonotic, dogs may serve as reservoirs or sentinels for these zoonotic pathogens [3, 4]. Additionally, dog population dynamics, such as birth and mortality rates, as well as human-driven movement, pose unique challenges when attempting to control pathogens such as rabies and canine distemper viruses via vaccination campaigns [5].

One of the most common ticks found on dogs in Africa is the brown dog tick (*Rhipicephalus sanguineus* sensu lato [s.l.], in particular *R. linnaei*), which can transmit numerous important pathogens [3, 6, 7]. For example, *Ehrlichia canis* is the causative agent of canine monocytotropic ehrlichiosis, which may be subclinical or cause multisystemic effects, including fever, anorexia, hemorrhagic tendencies (dermal petechiae and/or ecchymoses), and ophthalmological lesions [8]. *Hepatozoon canis* causes canine hepatozoonosis, which can be asymptomatic or associated with extreme lethargy, cachexia, and anemia [9]. *Babesia vogeli* is one of the causative agents of babesiosis in dogs but typically results in only moderate disease or nonclinical infection [10]. Finally, *Anaplasma platys* causes infectious cyclic thrombocytopenia (ICT) in dogs but rarely in humans. *Rhipicephalus sanguineus* s.l. is the suspected primary vector of *A. platys* [7].

Currently, there are few data on tick-borne pathogens in domestic dogs in Chad. These mixed-breed dogs live outdoors in rural villages and are primarily free-roaming and free-foraging, but some are intermittently fed by humans. Few dogs receive regular veterinary or preventative care. Dogs are commonly used to protect livestock and help with hunting. Due to their free-roaming nature, they are exposed to a wide variety of wildlife, livestock, habitats, vectors (i.e. ticks), and pathogens, and their regular interactions with humans can result in both direct and indirect pathogen transmission. Previous studies by this group on domestic Chadian dogs revealed high rates of tick infestation [11], a lack of heartworm (*Dirofilaria immitis*) infections, and infections with a *Brugia* sp. that was previously unreported in dogs in Central Africa [12]. To further evaluate the health of these dogs, a survey was conducted of selected tick-borne pathogens of known significance to dog health in Africa. Specifically, this study aimed to conduct surveillance for tick-borne pathogens among domestic dogs in Chad and determine (1) the prevalence of antibodies against *Anaplasma* spp., *Borrelia burgdorferi*, and *Ehrlichia* spp.; (2) the prevalence of *Hepatozoon* spp., *E. canis*, *A. platys*, and *Babesia* spp.; and (3) associations between these pathogens and demographic, spatial, and temporal factors.

## Results

A total of 428 dogs were sampled in May 2019. Of these initial 428 dogs, 314 were again sampled in November 2019, and 257 of these second round 314 were again sampled in June 2020.

### Serologic testing with the IDEXX SNAP 4Dx test

**Fig. 1:**
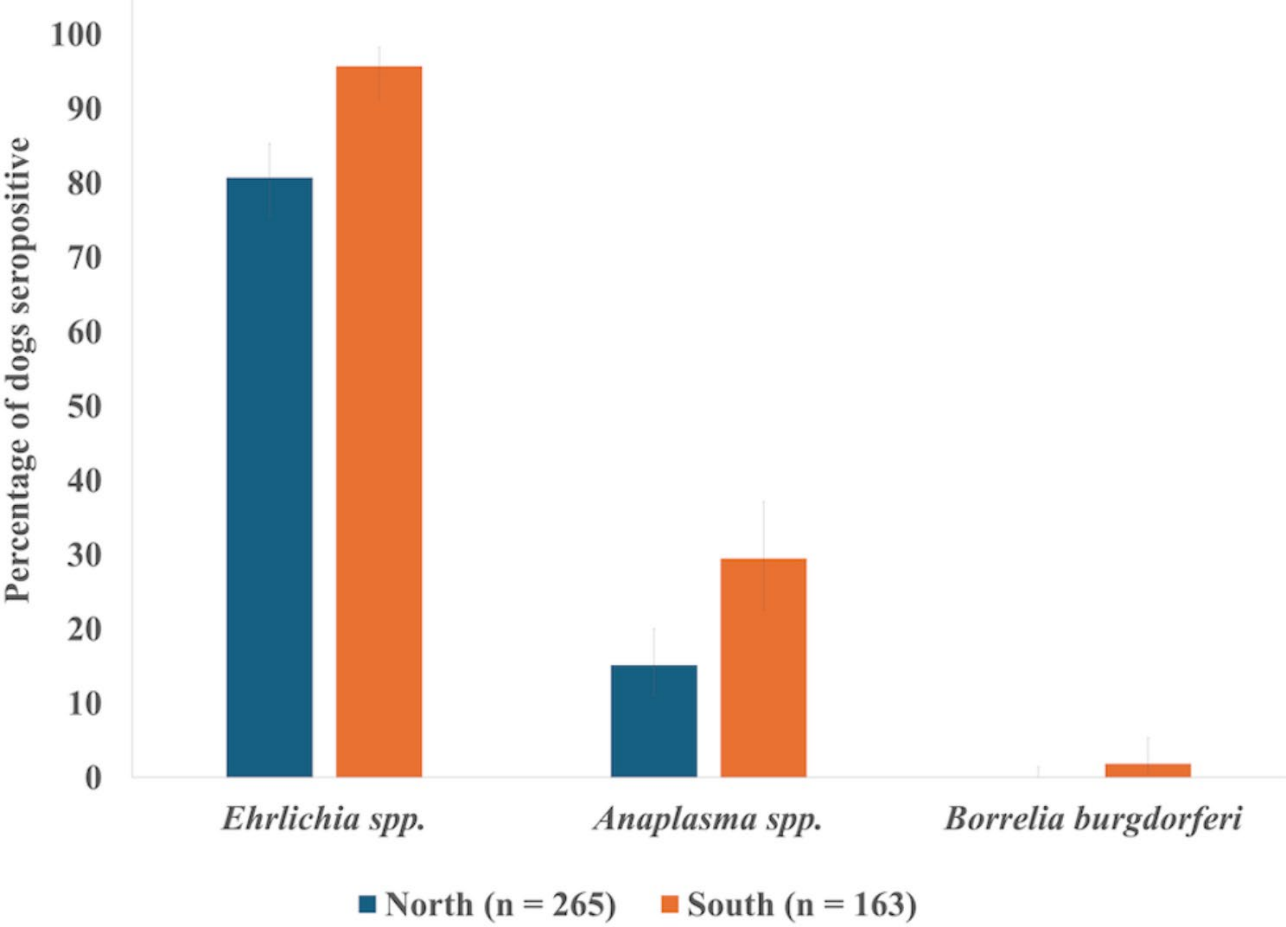
Percent of dogs from Chad, Africa (2019–2020), in the northern and southern regions of the study area seropositive for *Ehrlichia* spp., *Anaplasma* spp., and *Borrelia burgdorferi* according to the 4Dx SNAP test. Error bars indicate 95% confidence intervals

Of the 428 dogs that were tested with the SNAP 4Dx test in May 2019, 86% (*n* = 370, 95% CI = 83–90%) were positive for antibodies to *Ehrlichia* spp., 21% (*n* = 88, 95% CI = 17–25%) were positive for antibodies to *Anaplasma* spp., and 0.7% (*n* = 3, 95% CI = 0.1–2%) were positive for antibodies to *B. burgdorferi* (Table 1; Fig. 1). The results of *D. immitis* antigen testing and filarial worm molecular testing have been previously reported [12]. Of these 428 dogs, 0.7% (*n* = 3, 95% CI = 0.1–2%) were positive for all four tests; 6.3% (*n* = 27, 95% CI = 4–9%) had positive results for *Ehrlichia* spp., *Anaplasma* spp., and *D. immitis;* 19% (*n* = 80, 95% CI = 15–23%) had positive results for *Ehrlichia* and *D. immitis*; and 13% (*n* = 57, 95% CI = 10–17%) had positive results for *Ehrlichia* spp. and *Anaplasma* spp.

**Table 1:** Number of dogs seropositive for each of the three pathogen groups based on the IDEXX snap 4DX test

Pathogen	Total *n* = 428	Region		Sex^1^		Age in years^2^			
		North *n* = 265	South *n* = 163	Male *n* = 263	Female *n* = 164	1-1.5 *n* = 94	2-2.5 *n* = 151	3-3.5 *n* = 113	4–5 *n* = 70
*Anaplasma* spp.	88 (20.5)^3^	40 (15.1)	48 (29.4)	52 (19.8)	36 (22.0)	23 (24.5)	31 (20.5)	24 (20.5)	10 (14.3)
*Borrelia burgdorferi*	3 (0.7)	0	3 (1.8)	1 (0.4)	2 (1.2)	0	2 (1.3)	1 (0.9)	0
*Ehrlichia* spp.	370 (86.4)	214 (80.8)	156 (95.7)	224 (85.2)	146 (89.0)	75 (79.8)	132 (87.4)	100 (88.5)	63 (90)

^1^Sex was unknown for one dog

^2^Dogs were aged to the nearest 0.5 years

^3^Percentages are provided in parentheses

Generalized linear regression models revealed that geographic region of origin within the study area was a significant predictor of dogs being seropositive for *Ehrlichia* spp., with dogs in southern regions being more likely to test positive than those in northern regions (OR = 6.2, 95% CI = 2.6–14.8, *p* < 0.0001; Table 1; Fig. 1). Region of origin within the study area was also a significant predictor of *Anaplasma* spp. seropositivity, with dogs in southern regions more likely to have a positive result than dogs in the northern regions (OR = 2.4, 95% CI = 1.5–3.8, *p* = 0.0004; Table 1; Fig. 1). Neither sex nor age was a significant predictor of dogs testing positive for antibodies against these pathogens (Table 1). Three dogs that were seropositive for *B. burgdorferi* were from the southern region (two from Dankolo and one from Kaimamba) and included two females and one male (Table 1).

### Molecular testing with PCR

**Fig. 2:**
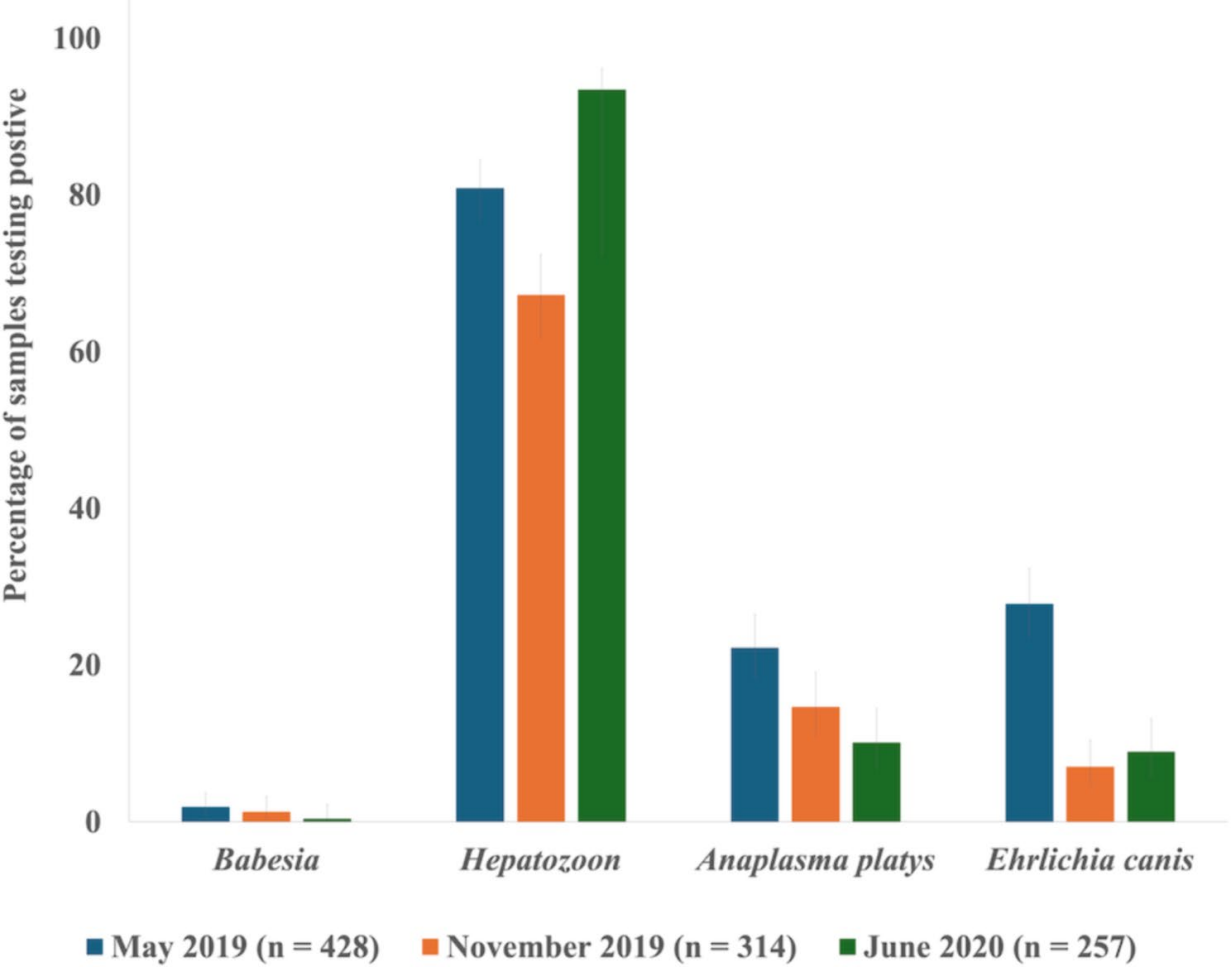
Percent of blood samples from domestic dogs in Chad, Africa, testing positive according to PCR for each of the four pathogens across the three time points in 2019 and 2020. The error bars show 95% confidence intervals

Based on PCR testing, four different pathogens were detected: *Hepatozoon* spp., *B. vogeli*, *E. canis*, and *A. platys* (Table 2; Fig. 2). The most commonly detected pathogen was *Hepatozoon* spp. Due to the large number of positive samples, only 83 of the 797 *Hepatozoon*-positive samples were randomly selected and sequenced; all were 98–100% similar to numerous *H. canis* sequences in GenBank. Similar prevalence levels of *E. canis* and *A. platys* were detected (Fig. 2). A random subset of the *E. canis-* and *A. platys*-positive samples (24 out of 164 and 33 out of 166, respectively) was sequenced and confirmed to be the expected pathogens. Thirteen samples across the entire study period were positive for *Babesia* spp., and sequence analysis of all amplicons confirmed infection with *B. vogeli*.

**Table 2:** Number of dogs whose blood samples were PCR-positive for each of the four pathogens at each of the three time points

May 2019		Region		Sex^1^		Age in years^2^			
Pathogen	Total *n* = 428	North *n* = 265	South *n* = 163	Male *n* = 263	Female *n* = 164	1-1.5 *n* = 94	2-2.5 *n* = 151	3-3.5 *n* = 113	4–5 *n* = 70
*Anaplasma platys*	94 (22.0)^3^	54 (20.4)	40 (24.5)	55 (20.9)	39 (23.7)	26 (27.6)	42 (27.8)	19 (16.8)	7 (10.0)
*Babesia vogeli*	8 (1.9)	0	8 (4.9)	4 (1.5)	4 (2.4)	2 (2.1)	5 (3.3)	0	1 (1.4)
*Ehrlichia canis*	119 (27.8)	56 (21.1)	63 (38.6)	84 (31.9)	35 (21.3)	25 (26.6)	43 (28.5)	33 (29.2)	18 (25.7)
*Hepatozoon canis*	346 (80.8)	194 (73.2)	152 (93.2)	214 (81.37)	131 (79.9)	75 (79.8)	123 (81.5)	94 (83.2)	54 (77.1)

**November 2019**		**Region**		**Sex** ^**1**^		**Age in years** ^**2**^			
**Pathogen**	**Total** ***n*** = **314**	**North** ***n*** = **185**	**South** ***n*** = **125**	**Male** ***n*** = **191**	**Female** ***n*** = **119**	**1-1.5** ***n*** = ***66***	**2-2.5** ***n*** = **106**	**3-3.5** ***n*** = **85**	**4–5** ***n*** = **54**

*Anaplasma platys*	46 (14.6)	38 (20.5)	8 (6.4)	28 (14.7)	18 (15.1)	20 (30.3)	14 (13.2)	7 (8.2)	5 (9.3)
*Babesia vogeli*	4 (1.3)	0	4 (3.2)	1 (0.5)	3 (2.5)	0	2 (1.9)	2 (2.4)	0
*Ehrlichia canis*	22 (7.0)	17 (9.2)	5 (4)	14 (7.3)	8 (6.7)	3 (4.5)	8 (7.5)	7 (8.2)	4 (7.4)
*Hepatozoon canis*	211 (67.2)	161 (87.0)	50 (40)	137 (71.7)	73 (61.3)	50 (75.8)	67 (63.2)	54 (63.5)	40 (74.1)

**June 2020**		**Region**		**Sex**		**Age in years** ^**2**^			
**Pathogen**	**Total** ***n*** = **257**	**North** ***n*** = **159**	**South** ***n*** = **98**	**Male** ***n*** = **157**	**Female** ***n*** = **97**	**1-1.5** ***n*** = **51**	**2-2.5** ***n*** = **84**	**3-3.5** ***n*** = **77**	**4–5** ***n*** = **42**

*Anaplasma platys*	26 (10.1)	19 (11.9)	7 (7.1)	16 (10.2)	10 (10.3)	8 (15.7)	7 (8.3)	8 (10.4)	3 (7.1)
*Babesia vogeli*	1 (0.4)	0	1 (1.0)	0	1 (1.0)	0	0	1 (1.3)	0
*Ehrlichia canis*	23 (9.0)	13 (8.2)	10 (10.2)	16 (10.2)	6 (6.2)	4 (7.8)	8 (9.5)	9 (11.7)	1 (2.4)
*Hepatozoon canis*	240 (93.4)	147 (92.5)	93 (94.9)	147 (93.6)	91 (93.8)	45 (88.2)	82 (97.6)	70 (90.9)	41 (97.6)

^1^Sex was unknown for one dog sampled in May and November 2019

^2^Dogs were aged to the nearest 0.5 years

^3^Percentages are provided in parentheses

Dog sex, geographic region, sampling time, and season were significant predictors of the presence of *Hepatozoon* spp. The best mixed-effects generalized linear regression model included the interaction between region and sampling time (Table 3). According to this model, dogs in the northern region were more likely to test positive for *Hepatozoon* spp. in November 2019 and June 2020 than in May 2019. Dogs in the southern region were more likely to test positive in May 2019 and June 2020 than in November 2019 (Table 4).

**Table 3:** AICc table of generalized linear models predicting the detection of *Hepatozoon* spp. in Chadian dogs in 2019 and 2020 based on region of origin (northern vs. southern), time point of testing, season of testing, and dog age and sex

Model	K^1^	AICc^2^	ΔAICc^3^	ω_i_^4^
Region * time point	7	833.5	0.00	0.504
Sex + region * time point	9	833.5	0.02	0.496
Sex + region + time point	7	940.7	107.23	0.000
Region + time point	5	941.7	108.22	0.000
Sex + time point	6	943.3	109.82	0.000
Time point	4	945.4	111.97	0.000
Sex + region + season	6	961.8	128.33	0.000
Region + season	4	961.8	128.36	0.000
Sex + season	5	964.2	130.73	0.000
Season	3	965.4	131.99	0.000
Sex + region	5	1001.8	168.33	0.000
Region	3	1002.3	168.89	0.000
Sex	4	1004.8	171.32	0.000
Null	2	1006.7	173.22	0.000

^1^K = number of parameters

^2^AIC_c_ = second-order Akaike information criterion

^3^ΔAIC_c_ = difference in AIC_c_ between ranked models

^4^ω_i_ = Akaike weight

**Table 4:** Odds ratios with 95% confidence intervals for the detection of *Hepatozoon* spp. based on the top-ranked linear regression model incorporating the interaction of region and time point of sampling. Only significant pairwise comparisons are shown

Level	vs.	Odds Ratio	95% CI lower limit	95% CI upper limit	*P*-value
North May 2019	South November 2019	5.39	2.34	12.41	< 0.0001
South May 2019	North May 2019	6.11	2.10	17.77	< 0.0001
South May 2019	South November 2019	32.91	9.62	112.57	< 0.0001
North November 2019	North May 2019	2.60	1.19	5.65	0.0063
North November 2019	South November 2019	13.98	5.07	38.61	< 0.0001
North June 2020	North May 2019	4.95	1.84	13.30	0.0001
South June 2020	North May 2019	7.47	1.80	30.98	0.0008
North June 2020	South November 2019	26.65	7.93	89.55	< 0.0001
South June 2020	South November 2019	40.32	8.59	188.46	< 0.0001

The dog age and time point of sampling were found to be significant predictors of *A. platys* detection, and the best model included the additive effects of these two factors (Table 5). According to this model, younger dogs were more likely to test positive, and dogs sampled in May 2019 were more likely to test positive for *A. platys* than were those sampled in November 2019 (OR = 1.65, 95% CI = 1.02–2.70, *p* = 0.0395) and June 2020 (OR = 2.54, 95% CI = 1.42–4.55, *p* = 0.0005).

**Table 5:** AICc table of generalized linear models predicting the detection of *Anaplasma platys* in Chadian dogs in 2019 and 2020 based on region of origin (northern vs. southern), time point of testing, season of testing, and dog age and sex

Model	K^1^	AICc^2^	ΔAICc^3^	ω_i_^4^
Age + time point	5	863.2	0.00	0.999
Age	3	876.3	13.10	0.001
Time point	4	884.8	21.63	0.000
Null	2	899.3	36.09	0.000

^1^K = number of parameters

^2^AIC_c_ = second-order Akaike information criterion

^3^ΔAIC_c_ = difference in AIC_c_ between ranked models

^4^ω_i_ = Akaike weight

The significant predictors of *E. canis* infection were dog sex, region, time point of sampling, and season. The top model included the additive effects of dog sex and the interaction between region and time point of sampling (Table 6). In general, based on this model, there were greater odds of *E. canis* being detected in both regions in May 2019. There were no significant pairwise comparisons according to sex (Table 7). Dog sex, age, geographic region, timing of sampling, and season were not significant predictors of *Babesia* spp. infection.

**Table 6:** AICc table of generalized linear models predicting the detection of *Ehrlichia canis* in Chadian dogs in 2019 and 2020 based on region of origin (northern vs. southern), time point of testing, season of testing, dog age, and dog sex

Model	K^1^	AICc^2^	ΔAICc^3^	ω_i_^4^
Sex + region * time point	9	804.5	0.00	0.788
Region * time point	7	807.2	2.73	0.201
Sex + region + time point	7	813.6	9.13	0.008
Region + time point	5	816.4	11.97	0.002
Sex + time point	6	819.9	15.40	0.000
Time point	4	821.5	17.00	0.000
Sex + region + season	6	853.3	48.88	0.000
Region + season	4	856.1	51.66	0.000
Sex + season	5	859.5	55.02	0.000
Season	3	860.8	56.31	0.000
Sex + region	5	887.7	83.24	0.000
Region	3	890.6	86.11	0.000
Sex	4	892.9	88.44	0.000
Null	2	894.3	89.81	0.000

^1^K = number of parameters

^2^AIC_c_ = second-order Akaike information criterion

^3^ΔAIC_c_ = difference in AIC_c_ between ranked models

^4^ω_i_ = Akaike weight

**Table 7:** Odds ratios with 95% confidence intervals for the detection of *Ehrlichia canis* based on the top-ranked linear regression model incorporating the effects of dog sex and the interaction between region and time point of sampling. Only significant pairwise comparisons are shown

Level	Vs	Odds Ratio	95% CI lower limit	95% CI upper limit	*P* value
South May 2019	North May 2019	2.99	1.35	6.59	0.0011
North May 2020	North November 2019	3.02	1.22	7.48	0.0067
North May 2021	South November 2019	7.13	1.70	29.90	0.0013
North May 2022	North June 2020	3.49	1.29	9.46	0.0047
South May 2019	North November 2019	9.03	3.16	25.82	< 0.0001
South May 2020	South November 2019	21.29	4.90	92.54	< 0.0001
South May 2021	North June 2020	10.43	3.37	32.26	< 0.0001
South May 2022	South June 2020	7.58	2.27	25.35	< 0.0001

**Fig. 3:**
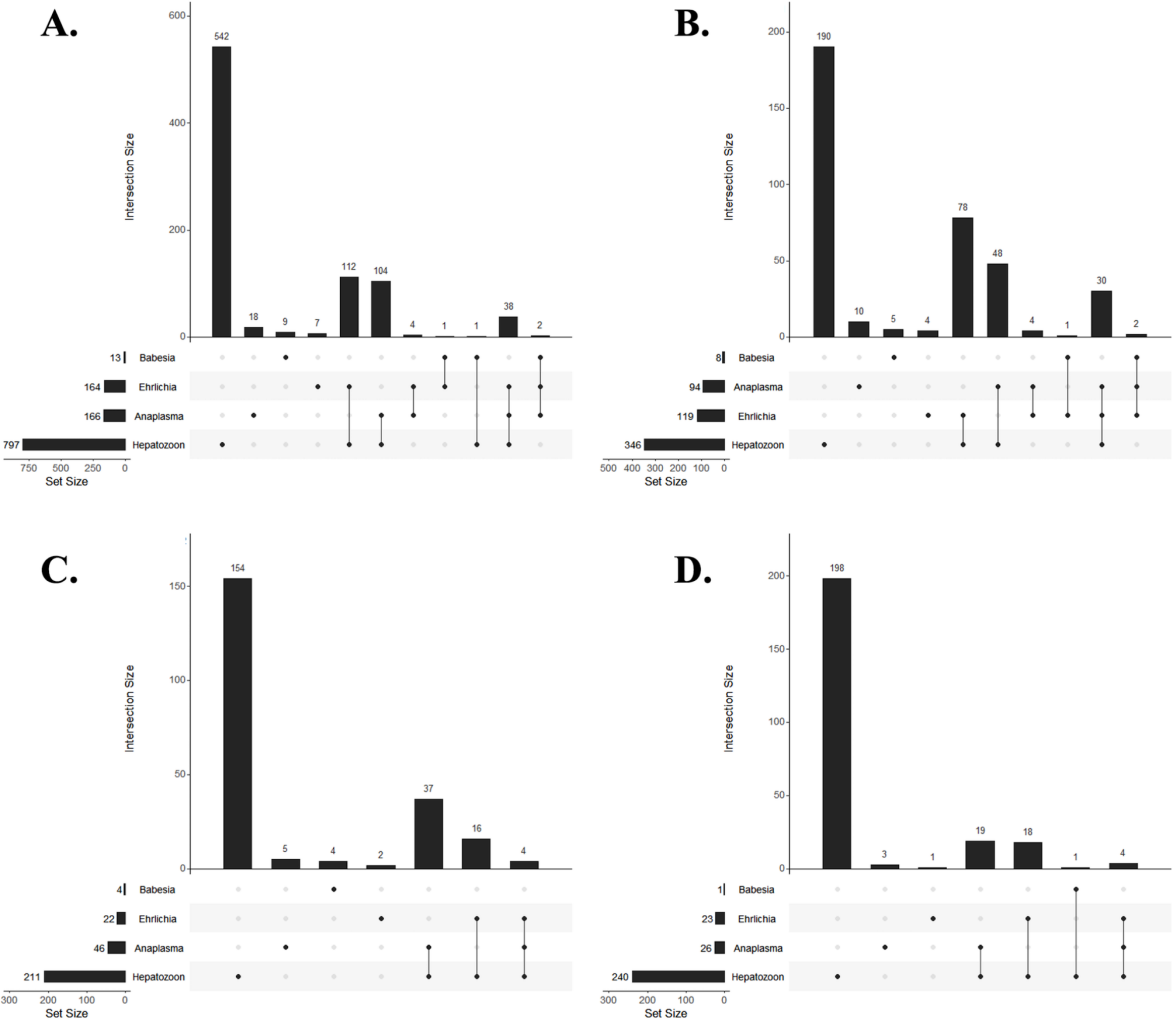
Frequency of blood samples from domestic dogs in Chad, Africa, testing positive for each of four pathogens, as determined via PCR, individually and in combination, in 2019 and 2020. (**A**) Data combined across all three time points; (**B**) May 2019; (**C**) November 2019; (**D**) June 2020

PCR revealed that 26% of the 999 samples tested (*n* = 262, 95% CI = 24–29%) had two or three pathogens detected (Fig. 3). The most common coinfection overall was *Hepatozoon* spp. with *E. canis* (Fig. 3A), and compared with those in November 2019 and May 2020, coinfections were most common, with the highest number of pathogen combinations occurring in May 2019 (Fig. 3B-D). Out of all the samples, 4% (*n* = 40, 95% CI = 3–5%) were positive for three pathogens; of these 40 samples, 38 were positive for *A. platys*, *E. canis*, and *Hepatozoon* spp., and two were positive for *A. platys*, *E. canis*, and *B. vogeli*. The latter combination was found only in May 2019. Out of all the samples, 22% (*n* = 222, 95% CI = 20–25%) were positive for two pathogens; of these 222 samples 112 were positive for *E. canis* and *Hepatozoon*; 104 were positive for *A. platys* and *Hepatozoon* spp.; four were positive for *A. platys* and *E. canis*; one was positive for *B. vogeli* and *E. canis*; and one was positive for *B. vogeli* and *Hepatozoon* (Fig. 3A). Considering longitudinal trends, no dogs were positive for *B. vogeli* at multiple time points; however, 33% of the 428 dogs sampled (*n* = 142, 95% CI = 29–38%) were positive for *Hepatozoon* twice, and 30% (*n* = 128, 95% CI = 26–34%) were positive at all three time points. Fewer dogs were positive for *A. platys* twice (*n* = 20, 5%, 95% CI = 3–7%) or at all three time points (*n* = 2, 0.5%, 95% CI = 0.06–1.7%), while 5% of dogs were positive for *E. canis* twice (*n* = 20, 95% CI = 3–7%) and 0.2% (*n* = 1, 95% CI = 0.1–1.3%) were positive at all three time points. Multiple positive detections over time may represent persistent infection or re-infection.

## Discussion

Tick-borne pathogens represent an important One Health issue, as many can cause disease in domestic and agricultural animals, wildlife, and humans. The present study found evidence of exposure to and/or infection with numerous tick-borne pathogens in dogs from Chad. Of these pathogens *A. platys*, *B. burgdorferi*, and *Ehrlichia* spp., are, or have the potential to be, zoonotic, and many of the tick species found on Chadian dogs also infest humans [13–16]. Additional studies are needed in Chad to monitor the prevalence and transmission of these pathogens, specifically, to understand the risks they pose to the health of domestic animals and humans.

Similar to other studies of African domestic dogs, *Hepatozoon* spp., specifically *H. canis*, was the most common pathogen detected, with a 40–94% prevalence depending on the region and time point of sampling (e.g [3, 17–19]). A multi-country study revealed a commensurately high prevalence of *H. canis* (average of 59%; Tanzania: 67–77%; Kenya: 54–85%; Uganda: 86–98%; Nigeria: 26–56%; Ghana: 46–68%; Namibia: 9–29%) [3]. Other studies also found a high prevalence in Sudan (42%), Ghana (40%), and Nigeria (41%) [17–19]. This characteristically high prevalence with wide distribution has been attributed to a large number of known vectors, including *Rhipicephalus* spp., and the potential for vertical transmission to puppies [20–22].

The detection of antibodies against *Ehrlichia* spp. and the molecular detection of *E. canis* were not surprising, as this pathogen has been reported in dogs from Chad and other African countries. Moreover, most dogs in this study were infested with *R. sanguineus* s.l [11], the primary vector of *E. canis* [3, 16–19, 23–29]. The 86% seroprevalence of *Ehrlichia* spp. in Chadian dogs in this study was considerably greater than that in a previous study in Chad (5/18 clinically normal military dogs, 28%) and against comparable studies in Ghana (21–35%), Sierra Leone (40%), and Nigeria (32–54%); however, the data in this study were consistent with those of a study from Senegal (89%) [3, 18, 24–26]. Generally, the prevalence of *E. canis* antibodies in southern and eastern African countries was lower (e.g., Tanzania: 29–32%; Kenya: 15–22%; Uganda 4–10%; Namibia: 25–40%) [16]; however, variation does exist, and higher prevalence rates have been reported (e.g., 73% in Zimbabwe, 96% in Sudan, and 87% among sick dogs in Namibia) [26–28]. The PCR prevalence of *E. canis* in Chadian dogs ranged from 4 to 39%, depending on the region and time point of sampling, similar to the findings of studies in numerous other sub-Saharan African countries, including neighboring Nigeria [19, 29]. While 86.4% of dogs were seropositive for *Ehrlichia* spp. in May 2019, only 27.8% were confirmed to be actively infected with *E. canis* at that time. A similar trend was noted in dogs from Zimbabwe, as well as several other southern and sub-Saharan African countries [3, 28]. This can be explained by dogs having been infected previously and cleared the infection but still having antibodies present in the blood. Alternatively, these animals may have been infected by an *Ehrlichia* spp. other than *E. canis*, e.g., with *E. ewingii*, *E. ruminantium*, *E. chaffeensis*, and potentially new species of *Ehrlichia* also reported in West Africa [1]. Variation in prevalence similar to that documented in this study has been observed among other western and sub-Saharan African countries, including 20% of dogs from Ghana, 7.3% of dogs from the Ivory Coast, 12.7% of dogs from Nigeria, and 6.4% of dogs from Algeria [18, 19, 30, 31].

Canine cyclic thrombocytopenia, caused by *A. platys*, is a significant disease of dogs in many regions of the world, and similar to *E. canis*,* R. sanguineus* s.l. is a suspected vector [1, 6, 7]. This pathogen is also a rare zoonosis [13]. This study’s finding of 21% seroprevalence for *Anaplasma* spp. among dogs in Chad is comparable to that in other countries in southern and sub-Saharan Africa, e.g., Ghana: 0–30%; Sierra Leone: 19%; Kenya: 8–10%; Nigeria: 4–20%; Tanzania: 20–21%; Uganda: 4–24%; and Namibia: 8–23% [3, 18, 25]. In Zimbabwe, 10% of 225 samples were seropositive [28]. Interestingly, in May 2019, only 20.5% of the dogs were seropositive for *Anaplasma* spp., while 22% of the dogs were PCR positive for *A. platys*. This difference is likely due to recently infected animals not having yet mounted an antibody response, as the response is first detectable 16 days after infection [32]. In the current study, the prevalence of *A. platys* via PCR varied from 6 to 24%, depending on the region and time point of sampling, which is similar to the findings in nearby countries in sub-Saharan Africa (Kenya (10–23%), Ghana: 10%; Ivory Coast: 1.5% and 0–30%; Gabon: 1.2%; and Nigeria: 6.6%), as well as northern Africa (Algeria: 5.4%) [7, 18, 19, 30, 31].

A small number of the dogs sampled in this study (*n* = 13) were positive for *B. vogeli*, one of three closely related canine species including *B. canis*, *B. rossi*, and *B. vogeli* that are distinguished by biological characteristics and molecular methods [10, 33]. For example, *B. rossi* is transmitted by *Haemaphysalis* spp., and infection is typically fatal, while *B. vogeli* is transmitted by *R. sanguineus* s.l. and is considered the least pathogenic [10]. The prevalence of *Babesia* spp. in dogs in sub-Saharan Africa varies considerably from 0 to 12% depending on country and rural vs. urban area [3]. In countries neighboring Chad, 9% of dogs from Sudan were positive for *Babesia* spp. (five with *B. rossi* and two with *B. vogeli*) [17], and both *B. rossi* and *B. vogeli* have been detected in dogs in Nigeria, but *B. rossi* was more common [19, 34, 35]. The lack of *B. rossi* in the dogs in this study may be due to the low number (*n* = 14) of dogs infested with *Haemaphysalis leachii* [16]. Additional studies to determine the distribution and factors related to the presence and intensity of *H. leachii* are needed to better understand the risk of severe babesiosis to the health of dogs in Chad.

The high number of dogs that were positive for antibodies against both *Anaplasma* spp. and *Ehrlichia* spp. is not surprising, given that both pathogens are transmitted by *R. sanguineus* s.l [6, 25]. Among 53 dogs from Sierra Leone tested with the SNAP 4Dx test, 9.4% were positive for both *Ehrlichia* spp. and *Anaplasma* spp. antibodies, and 5.7% were positive for *Ehrlichia* spp. antibodies, *Anaplasma* spp. antibodies, and *D. immitis* [25]. Furthermore, antibodies against these pathogens have been shown to persist for months to years [36, 37]. Coinfections with two or three pathogens were detected by PCR in 24.2% of the samples in the current study, with the most common combination being *Hepatozoon* spp. and *Ehrlichia canis*, and 4.0% of the samples had three pathogens detected. This finding is similar to that of a multinational study of African dogs, in which 30.9% of the dogs were coinfected with at least two pathogens, the most common combination (10.1%) being *H. canis* and *E. canis*, and 5.1% of the dogs had three or four pathogens in their blood [3]. Coinfections are not surprising given that these pathogens share at least one tick vector group, *Rhipicephalus sanguineus* s.l., and these ticks were commonly detected on dogs in this study.

There were several significant spatiotemporal and demographic factors associated with the detection of exposure or infection with multiple pathogens included in this study. Dogs in the southern region were more likely to be seropositive for *Ehrlichia* spp. and *Anaplasma* spp. and to be infected with *Hepatozoon* spp. and *E. canis* than dogs in the northern region were. This may be explained by climate variation within Chad, with differences between regions that can impact tick populations: the northern study areas are more arid, and the southern region of Chad receives more rainfall [38]. For all three pathogens (*Hepatozoon*, *A. platys* and *E. canis*), the time point was a significant predictor of detection. In the northern region, *Hepatozoon* spp. were more likely to be detected later in the study (November 2019 and June 2020 > May 2019), whereas in the southern region, *Hepatozoon* spp. were more likely to be detected earlier in the study (May 2019 and June 2020 > November 2019). Overall, *A. platys* and *E. canis* were more likely to be detected earlier in the present study (May 2019 vs. November 2019 and June 2020). *Ehrlichia canis* infection typically occurs during the dry-hot season when the tick *Rhipicephalus sanguineus* s.l. is active [8]. It is also possible that the removal of ticks from the study dogs at the three time points may have reduced the pathogen prevalence at later time points, as ticks were no longer present to transmit the pathogens of interest; however, that only represented a few days throughout the year.

Dog age was a significant predictor for the detection of *A. platys* by PCR. The finding that younger dogs were more likely to be infected with *A. platys* agrees with the findings of previous work in Kenya and Ivory Coast that showed a 19.8% prevalence in dogs younger than one year, compared to 6.7% in adult dogs [7]. This supports the finding that dogs were more likely to be infected in May 2019 than at the two later time points of the study, as dogs were youngest at the first time point of the study. Furthermore, while infections with *A. platys* persist for months, dogs may clear infections after 100–150 days [32]. This is consistent with the finding that dogs were more often positive for *A. platys* at two consecutive time points (14 dogs) than at the first and third time points (six dogs) or at all three time points (two dogs).

The detection of antibodies against *B. burgdorferi* was unexpected based on the historical range of this pathogen in North America and Eurasia and its predominant association with *Ixodes* spp. ticks [15]. However, there are sporadic reports of this pathogen outside the expected range. In Africa, 1.4% of dogs in rural Kenya were seropositive for *Borrelia* spp [3], and a single dog in Egypt and an associated *R. sanguineus* s.l. tick tested positive via PCR [39]. Another study in Egypt detected *B. burgdorferi* via PCR in 23% of dogs (*n* = 26), 16% of cattle (*n* = 25), 58% of dog-associated *R. sanguineus* s.l. (*n* = 12), and 21% of bovine-associated *Hyalomma anatolicum excavatum* (*n* = 14) [40]. Although no *Ixodes* were found on any of the dogs in this study, the three *B. burgdorferi-* positive dogs were infested with *R. sanguineus* s.l. In addition to the true exposure of Chadian dogs to *B. burgdorferi*, there are other possible explanations for these findings. It is possible that these results represent cross-reaction with other *Borrelia* spp. or false positives. In Africa, relapsing fever group (RFG) *Borrelia* spp., such as *B. recurrentis* in countries east of Chad and *B. crocidurae* in countries north of Chad, have been reported, but rarely do RFG *Borrelia* cross-react with C6-based serologic tests [41, 42]. However, only a limited number of *Borrelia* species have been evaluated, so it is possible that some RFG *Borrelia* may cross-react. Additionally, a novel lineage of *Borrelia*, distinct from both the relapsing fever and Lyme disease groups, has been reported in *Amblyomma* spp. from Ethiopia and the Ivory Coast, including *A. variegatum*, a tick species found on dogs in Chad [16, 43, 44]. The cross-reactivity of this group with *B. burgdorferi* C6 assays is not known.

Aspects of this study limit the conclusions that can be drawn from the data. Importantly, ticks were opportunistically collected from dogs enrolled in an experimental therapeutic trial for the treatment and prevention of Guinea worms (*Dracunculus medinensis*) [13]. Therefore, sample size calculations and counts of total tick burden per dog were not performed, which limits the interpretability of the results. Additionally, there were only three time points of sample collection, and the SNAP 4DX tests were performed only at the first time point. More robust conclusions about prevalence trends could be drawn from data collected over many years, with multiple years of sampling during each season. Another limitation was that outwardly sick dogs were excluded based on the primary study criteria; therefore, analyzing data for associations between pathogen detection and clinical illness was not possible. Moreover, the number of dogs sampled decreased over time as dogs either died or moved with their owners away from the village where they were originally sampled. In addition, the ticks found on each dog were removed for subsequent testing at each time point, potentially reducing pathogen transmission and impacting prevalence estimates at later time points. Finally, animals testing positive for a pathogen multiple times over the course of the study may represent persistent infection or re-infection with that pathogen. This cannot be differentiated by the methods of this study, so these repeat positives were included in the statistical analysis to represent the probability of a given dog testing positive at each time point. However, a positive test for a pathogen at an earlier time point may influence the status of that dog at later points.

## Conclusions

In summary, this study found that many domestic dogs in Chad had evidence of exposure to and/or infection with multiple pathogens, including *E. canis*, *A. platys*, *B. burgdorferi*, *B. vogeli*, and *Hepatozoon* spp. (some confirmed to be *H. canis*). Given the high prevalence of several pathogens in dogs (*H. canis*,* E. canis*, and *A. platys*), veterinarians in Chad should consider tick-borne diseases in dogs that present with appropriate clinical signs or abnormalities. Dog owners should also be encouraged to use appropriate preventatives to limit exposure to ticks and other vectors. Given that some of these pathogens are known, or are suspected to be, zoonoses, this study presents a One Health approach to understanding pathogen dynamics in Chad and indicates that additional work is needed to understand the risks these pathogens pose to domestic animals, wildlife, and humans.

## Materials and methods

### Sample collection

**Fig. 4:**
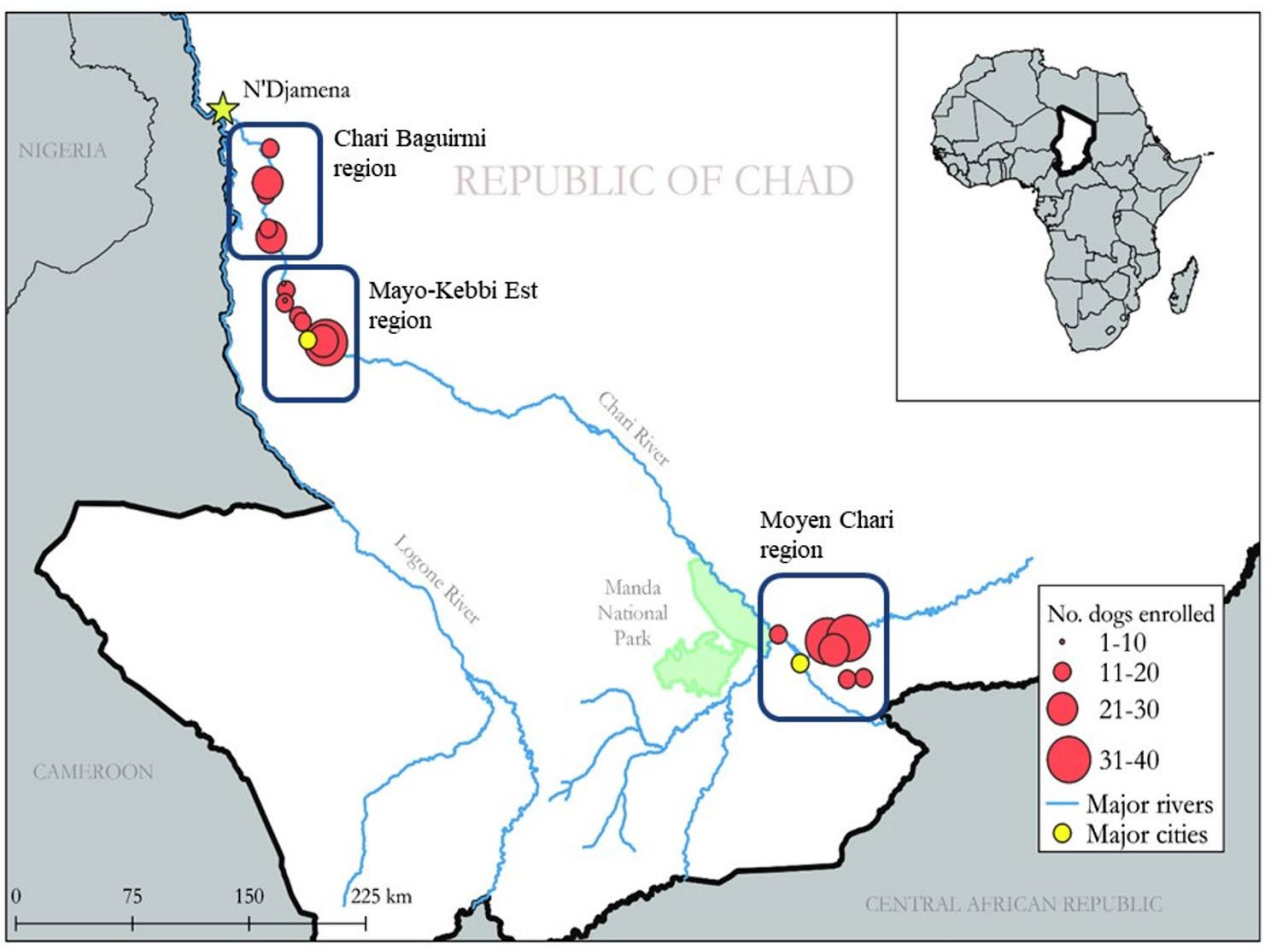
Map of the study area in Chad, Africa, showing the regions from which dogs were sampled. This map was previously published by Cleveland et al. (2022) under the terms “creative common attribution” (CC-BY) license (https://creativecommons.org/licenses/by/4.0/) and has not been modified from its original form

Blood samples were serially collected from the same individual domestic dogs in Chad, Africa, at three time points: May 2019, November 2019, and June 2020. In Chad, May and June are during the wet season, and November is the dry season. As part of a concurrent study [45], dogs were sampled in 23 villages from three regions (Moyen-Chari, Chari Baguirmi, and Mayo-Kebbi Est) (Fig. 4) based on the following criteria: owner approval for sample collection; dog age between one and five years; dogs lacking overt signs of significant illness; and dog demeanor allowing approach and restraint for sampling. The sex, age (estimated to the nearest 0.5 years), and village of origin of each dog were recorded.

Venipuncture sites were aseptically prepared with 70–90% EtOH, and a blood sample (~ 0.7 mL) was collected from the cephalic vein. Blood was placed in 3 mL EDTA vacutainer tubes (Becton, Dickinson and Company, Franklin Lakes, New Jersey, USA) and stored in a field cooler with an ice pack. Upon return to the field laboratory, ~ 125 µL of blood was transferred to Whatman™ FTA™ cards (Cytiva, Marlborough, Massachusetts, USA) for pathogen screening. In May 2019, whole blood was tested for antibodies against *Anaplasma* spp., *Ehrlichia* spp., and *Borrelia burgdorferi*, as well as *Dirofilaria immitis* antigens, using an IDEXX SNAP 4Dx test (IDEXX Laboratories, Portland, Maine, USA) per the manufacturer’s instructions. All animal procedures were reviewed and approved by the University of Georgia’s Institutional Animal Care and Use Committee (A2019 04–005), the Chad Ministry of Health, and the Institut de Recherche en Elevage pour le D´eveloppement (IRED), which is the research institution in charge of animal research in Chad and authorized by the Ministry of Livestock and Animal Production.

### DNA extraction and molecular assays

DNA was extracted from the FTA cards according to the manufacturer’s protocol using a commercially available DNA extraction kit (QIAamp DNA Investigator Kit, Qiagen, Valencia, CA, USA) [46]. DNA was screened for four pathogens using the PCR protocols in Table 8. Two different PCR protocols were used to detect *Babesia* spp. and *Hepatozoon* spp. based on different gene targets: 18 S PCR provided sequences for species-level identification, while ITS PCR produced amplicons of different sizes for the two genera, allowing genus-level differentiation based on the band location on the gel rather than requiring sequencing for every amplicon. For all the assays, the amplicons were purified from a 0.8% agarose gel stained with Gel Red (Biotium, Inc., Hayward, California, USA) using a commercial gel purification kit (Qiagen). Bidirectional Sanger sequencing was conducted by Genewiz (South Plainfield, New Jersey, USA), and the sequences were edited and assembled using Geneious 10.2.6 (Biomatters Limited, Auckland, New Zealand). The consensus sequences were subsequently used as queries for BLASTN searches against the National Center for Biotechnology Information (NCBI) GenBank nucleotide sequence database.

**Table 8:** PCR protocols used to screen blood samples from domestic dogs in Chad, Africa

Pathogen	Gene Target (size in bp)	Primers	Primer Sequence (5’➔ 3’)	Reference
*Babesia* spp. and *Hepatozoon* spp.	18 S rRNA (500 for *Babesia*, 600 for *Hepatozoon*)	Primary: 5.1 / B	CCTGGTTGATCCTGCCAGTAGT TGATCCTTCTGCAGGTTCACCTAC	[47, 48]
		Secondary: Babesia F / Babesia R	GTGAAACTGCGAATGGCTCA CCATGCTGAAGTATTCAAGAC	
	Internal Transcribed Spacer rRNA (ITS) (300 for *Babesia*, < 200 for *Hepatozoon*)	Primary: 15 C / 13B	CGATCGAGTGATCCGGTGAATTA GCTGCGTCCTTCATCGTTGTG	[50]
		Secondary: 15D / 13 C	AAGGAAGGAGAAGTCGTAACAAGG TTGTGTGAGCCAAGACATCCA	[51]
*Ehrlichia canis*	16 S rRNA (389)	Primary: ECC / ECB	AGAACGAACGCTGGCGGCAAGCC CGTATTACCGCGGCTGCTGGCA	[52]
		Secondary: ECA / HE3	CAATTATTTATAGCCTCTGGCTATAGG TATAGGTACCGTCATTATCTTCCCTAT	[52, 53]
*Anaplasma platys*	16 S rRNA (400)	Primary: ECC / ECB	AGAACGAACGCTGGCGGCAAGCC CGTATTACCGCGGCTGCTGGCA	[52]
		Secondary: PLA2 / GA1UR	TTTGTCGTAGCTTGCTATG- GAGTTTGCCGGGACTTCTTCT	[54, 55]

### Statistical analyses

The prevalence of each pathogen, with corresponding 95% confidence intervals (CIs), was calculated for dogs with positive results for each pathogen or group of pathogens on the SNAP 4Dx test and for dogs with positive PCR results for each pathogen at each time point. Generalized linear regression models (function glm in the R package stats [56]) were used to predict SNAP-positivity based on dog age (continuous variable) and sex, as well as geographic region of origin within the study area (south [Moyen-Chari region] or north [Chari Baguirmi and Mayo-Kebbi Est regions]). A series of mixed-effects generalized linear regression models were created using the function glmer in the R package lme4 [57] to predict the PCR results for each pathogen based on the fixed effects of dog age, sex, geographic region of origin, time point of sampling, season (May and June during the wet season and November during the dry season), and the random effect of dog ID to account for repeated sampling of the same dogs. Predictors with *p* > 0.2 in univariable models were included in a set of multivariable models examining the additive and interactive effects of significant predictors. Models were evaluated using an information theoretic approach [58]. Statistical analyses were performed in RStudio version 2022.07.0 [59], with statistical significance assessed at α = 0.05.

## Data Availability

All the data generated or analyzed in this study are included in the article, and any additional inquiries can be directed to the corresponding author.
